# Integrated PTR-ToF-MS, GWAS and biological pathway analyses reveal the contribution of cow’s genome to cheese volatilome

**DOI:** 10.1038/s41598-018-35323-5

**Published:** 2018-11-19

**Authors:** Sara Pegolo, Matteo Bergamaschi, Flavia Gasperi, Franco Biasioli, Alessio Cecchinato, Giovanni Bittante

**Affiliations:** 1 Department of Agronomy, Food, Natural Resources, Animals and Environment (DAFNAE), University of Padua, Viale dell’Università 16, 35020 Legnaro, Padua Italy; 2 Department of Food Quality and Nutrition, Research and Innovation Centre, Fondazione Edmund Mach (FEM), Via E. Mach 1, 38010 San Michele all’Adige, TN Italy

## Abstract

Volatile organic compounds (VOCs) are small molecules that contribute to the distinctive flavour of cheese which is an important attribute for consumer acceptability. To investigate whether cow’s genetic background might contribute to cheese volatilome, we carried out genome-wide association studies (GWAS) and pathway–based analyses for 173 spectrometric peaks tentatively associated with several VOCs obtained from proton-transfer-reaction mass spectrometry (PTR-ToF-MS) analyses of 1,075 model cheeses produced using raw whole-milk from Brown Swiss cows. Overall, we detected 186 SNPs associated with 120 traits, several of which mapped close to genes involved in protein (*e.g. CSN3*, *GNRHR* and *FAM169A*), fat (*e.g. AGPAT3*, *SCD5*, and *GPAM*) and carbohydrate (*e.g. B3GNT2*, *B4GALT1*, and *PHKB*) metabolism. Gene set enrichment analysis showed that pathways connected with proteolysis/amino acid metabolism (purine and nitrogen metabolism) as well as fat metabolism (long-term potentiation) and mammary gland function (tight junction) were overrepresented. Our results provide the first evidence of a putative link between cow’s genes and cheese flavour and offer new insights into the role of potential candidate loci and the biological functions contributing to the cheese volatilome.

## Introduction

Cheese quality depends on many related, interacting factors, ranging from compositional, functional, sensory and safety characteristics to nutritional, psychological, convenience, processing and economic factors^1^. Consumer acceptability of dairy products is highly dependent on sensory characteristics^2^, particularly flavour, an important determinant of quality. During the manufacture and ripening of cheese, enzymes from various sources (native milk enzyme, rennet, lactic acid bacteria, secondary microflora and exogenous enzyme preparations) are responsible for the breakdown of macronutrients (fat, proteins and lactose) into fatty acids, amino acids and lactic acid, the major precursors of volatile organic compounds (VOCs), which play a significant role in determining cheese flavour^3,4^.

Several studies aimed at characterizing the volatile fraction of various cheeses have been conducted^5–7^, most of them using solid-phase micro-extraction (SPME)-GC-MS equipment. Proton-transfer-reaction time-of-flight mass spectrometry (PTR-ToF-MS), however, is a more time-efficient and sensitive method for characterising the cheese VOC fingerprint^8,9^. Several factors (e.g. dairy system, herd, individual cow characteristics) have been shown to affect the cheese volatilome^9,10^ and evidence for the existence of an exploitable genetic variation in the cheese VOC profile has also recently been put forward^11^, suggesting there is potential to modify cheese flavour through selective breeding in order to improve cheese quality.

Genome-wide association studies (GWAS) have been widely used to disentangle the genomic architecture underlying complex traits in dairy cattle^12–14^. It has become common to couple GWAS with biological pathway analysis to extract biological information from the GWAS data and overcome the limitations of this method, such as the its reduced ability to detect small-effect loci and its poor replication^15–17^. The genomic and biological information thus acquired makes it possible to elucidate the genetic basis and molecular mechanisms underlying complex traits on the one hand, and, on the other hand, to increase the accuracy of genomic prediction when incorporated into prediction models^18,19^.

Herein, we investigated whether cow’s genetic background contributes to variability in the cheese volatilome and, therefore, might play a role in determining cheese flavour. The potential existence of a genomic control for VOC profile in cheese would be of considerable significance given the economic importance of cheese quality to the dairy industry. To our knowledge, there is no existing information on whether there is a relationship between the cow’s genome and the cheese VOC profile, nor on the biological functions that may be involved in regulating the cheese volatilome. The aim of this study, therefore, was i) to perform GWAS analyses for milk and cheese composition traits in dairy cows, and for cheese VOC profiles determined by proton-transfer-reaction time-of-flight mass spectrometry (PTR-ToF-MS), and ii) to carry out pathway analyses on the SNP markers, in order to identify genomic regions and biological mechanisms that contribute to the variability in cheese volatilome.

## Results

Descriptive statistics and genomic heritability estimates for milk and cheese composition are reported in Table 1. We found milk fat percentage to have a relatively low heritability (0.08), and confirmed protein percentage as being under strong genetic influence (0.40). Lactose percentage was moderately heritable (h^2^ = 0.22), while heritability estimates were, instead, close to 0 for the milk fat to protein ratio, and cheese fat and protein, which depend mainly on the cheese-making procedure. Table 2 shows the concentrations and heritabilities for some of the spectrometric peaks associated with the VOCs of model cheeses measured by PTR-ToF-MS. Among the tentatively identified spectrometric peaks, those associated with dimethylsulfone *m/z* 95.017 (0.22), alkyl fragment (terpenes) *m/z* 81.070 (0.15), butan-1-ol/pentan-1-ol, heptan-1-ol *m/z* 75.080 (0.14) and hexanal/nonanal *m/z* 83.086 (0.10) had moderate heritabilities. Among the unknown compounds, the peaks at *m/z* 85.029 (0.22), *m/z* 135.134 (0.18), *m/z* 66.063 (0.18), *m/z* 48.053 (0.17), *m/z* 44.980 (0.15), *m/z* 83.071 (0.15) and *m/z* 169.044 (0.15) had the highest heritabilities.

**Table 1:** Descriptive statistics and genomic heritability (*h*^2^) for milk and cheese composition.

Trait	Mean	SD	h^2^	#SNP^a^
Milk yield, kg/d	24.26	7.96	0.09	2
Milk composition				
Fat, %	4.19	0.67	0.08	5
Prot, %	3.71	0.42	0.40	2
Fat to prot	1.13	0.18	<0.01	1
Lactose, %	4.85	0.20	0.22	1
Cheese composition				
Fat, %	38.11	4.23	<0.01	2
Prot, %	27.08	4.04	<0.01	5

^a^#SNP: number of significant SNP (5 × 10^−5^) for each trait.

**Table 2:** Descriptive statistics and genomic heritability (*h*^2^) for some spectrometric peaks from PTR-ToF-MS analysis of model cheeses*.

Measured mass(m/z)	ppb		h^2^	#SNP^a^
	Mean	SD		
Volatile compounds				
44.980	4.64	0.66	0.15	1
48.053	10.03	0.82	0.17	5
49.011	6.82	0.67	0.08	—
49.028	5.28	0.73	0.10	2
63.044	8.02	0.49	0.08	3
66.063	8.61	0.86	0.18	5
75.027	5.47	0.96	0.08	3
75.080	7.70	1.14	0.14	5
78.001	4.23	0.89	0.05	3
79.075	6.17	0.64	0.07	2
81.070	5.37	0.46	0.15	4
82.945	4.48	0.70	0.13	3
83.071	7.44	0.95	0.15	3
83.086	6.13	0.69	0.10	3
84.075	4.41	0.58	0.09	3
84.942	4.29	0.63	0.08	5
85.029	4.46	0.40	0.20	3
91.059	8.47	0.81	0.15	2
92.061	6.64	0.97	0.05	2
93.090	10.11	1.37	0.13	4
93.432	4.54	0.46	0.07	3
94.039	5.43	0.90	0.13	2
94.095	6.92	1.37	0.11	3
95.004	4.72	0.65	0.05	1
95.017	5.04	0.71	0.22	3
95.034	5.22	0.64	0.10	2
95.096	5.44	0.98	0.14	2
105.039	4.53	0.44	0.07	5
109.070	6.30	0.70	0.06	2
111.104	5.08	0.94	0.12	2
113.029	4.64	0.48	0.06	1
119.072	5.54	0.66	0.11	5
119.089	6.44	1.00	0.13	2
121.122	5.17	0.82	0.13	5
123.047	4.56	0.35	0.05	2
123.076	4.56	0.38	0.09	3
123.117	4.82	0.64	0.06	2
127.073	4.49	0.38	0.08	1
129.064	4.37	0.37	0.13	—
129.127	4.95	0.69	0.06	—
133.102	4.45	0.38	0.06	—
133.123	5.60	0.69	0.07	3
135.102	6.69	0.62	0.07	—
135.134	5.80	0.75	0.18	3
137.101	4.71	0.50	0.12	4
137.132	5.28	0.39	0.16	2
139.076	4.58	0.35	0.06	2
139.134	4.12	0.54	0.09	2
149.045	6.08	0.97	0.16	6
157.159	4.33	0.46	0.08	1
163.096	6.55	1.25	0.10	1
169.044	5.92	0.92	0.15	5
171.173	4.87	0.76	0.11	3
173.153	5.20	0.48	0.09	1
189.184	4.23	0.50	0.07	—
191.163	4.35	0.32	0.07	—
201.184	4.34	0.41	0.07	2

*Only the peaks with h^2^ ≥ 0.05 are presented.

^a^#SNP: number of significant SNP (5 × 10^−5^) for each trait.

Results of the GWAS analyses of milk and cheese composition and cheese VOCs are summarised in Table 3 and Supplementary Table S1. Overall, we detected 186 significant SNPs (*P* < 5E-05) across all *Bos taurus* autosomes (BTAs), which were associated to 120 traits. One SNP had an unknown position on the genome, which was significantly associated with *m/z* 131.107 (*P* = 1.04E-05). Most of the significant associations were one SNP-one trait (80%).

**Table 3:** Summary results of the genome wide association analysis for spectrometric peaks from PTR-ToF-MS analysis of model cheeses.

BTA^a^	#SNP	Interval, Mbp	Top SNP	Top SNP P-value	Top SNP location, bp	Top SNP MAF	Trait(m/z)^b^
1	1	—	rs110470451	2.43E-05	6846585	0.42	61.062
1	1	—	rs109360740	2.50E-05	9601018	0.36	75.080, 48.053, 66.063, **84.075**, 95.096
1	1	—	rs110588394	4.87E-05	44569518	0.02	113.029
1	2	98.34–100.38	rs41585810	8.97E-06	100382840	0.07	91.059, **123.047**,137.101, 139.076
1	1	—	rs110086518	3.58E-05	119931878	0.12	55.055
1	1	—	rs110442512	3.07E-05	131684053	0.25	115.077
1	1	—	rs110224946	3.59E-05	136127682	0.47	51.044
1	2	146.59–147.58	rs109454192	3.79E-06	147582980	0.04	159.138, **83.052**
2	1	—	rs110662635	4.20E-05	24734064	0.34	43.054
2	1	—	rs43304641	4.95E-05	32154806	0.02	103.075
2	1	—	rs41573759	2.68E-05	52612052	0.04	93.432
2	1	—	rs41643281	4.93E-05	135167307	0.43	43.054
3	1	—	rs109805934	1.95E-05	26438136	0.45	105.071
3	1	—	rs110682053	2.83E-05	29461800	0.22	105.091
3	1	—	rs42824274	4.31E-05	47337477	0.15	63.044
3	3	79.73–81.77	rs43349836	2.87E-05	81774972	0.01	33.034, 51.044, **93.037**
3	1	—	rs29011217	3.87E-05	95279013	0.22	109.07, 95.034, **169.044**
3	1	—	rs43712201	4.87E-05	98450919	0.30	95.004
3	2	105.61–106.61	rs42791325	1.35E-05	105613230	0.46	95.034, **91.051**
4	1	—	rs110991247	6.18E-06	13148484	0.49	129.091, **155.144**, 173.153, 201.184
4	3	20.69–22.62	rs109900996	7.78E-06	22618815	0.02	113.098, **fat**_**MILK**_
4	1	—	rs42358265	1.18E-05	35447660	0.17	49.028,131.084
4	1	—	rs42435059	4.43E-06	39244447	0.01	fat_CHEESE_
4	2	70.42–70.45	rs110731593	3.13E-05	70419951	0.02	95.081
4	2	93.71–93.74	rs110600059	3.00E-05	93709495	0.27	106.077
4	1	—	rs43373704	1.84E-05	119552457	0.05	113.098
5	1		rs109438971	5.76E-06	27130695	0.05	92.061
5	1		rs43439308	4.73E-05	88311948	0.38	105.039
5	1		rs109173922	1.30E-05	110982640	0.33	105.091
5	1		rs110758831	4.06E-05	115561004	0.49	105.091
6	1	—	rs110587419	1.64E-05	37019972	0.05	MY
6	1	—	rs42005069	4.83E-05	55147990	0.21	63.044
6	2	73.25–73.82	rs41653762	4.50E-05	73254801	0.16	**149.045**, 123.076
6	9	81.65–88.07	rs110239739	1.32E-07	84689991	0.16	**prot**_**MILK**_, MY,163.096,85.029, 149.045, 93.037, 169.044, 75.027, 137.101, 123.047, 109.070, 91.059
6	1	—	rs41591081	3.41E-05	91840239	0.21	89.060
6	1	—	rs110986676	3.57E-05	99341521	0.14	116.078
7	1	—	rs43506578	4.65E-05	24599167	0.19	115.112
7	2	65.47–67.20	rs110580802	1.37E-05	67202130	0.09	119.107, **111.119**
7	1	—	rs42383787	2.74E-05	76668676	0.21	44.058, **61.062**
7	2	—	rs41574792	3.42E-05	105537981	0.31	57.070, **82.945**
8	1	—	rs43097793	3.75E-05	4503268	0.06	105.039
8	1	—	rs41654691	2.74E-05	49723924	0.11	137.132
8	2	68.44–69.53	rs41589887	3.13E-05	69525276	0.09	48.053, 135.134, **99.039**
8	1	—	rs110002748	1.94E-05	74997682	0.28	117.047
8	2	98.88–99.05	rs109263494	2.38E-05	98881581	0.01	**75.08**, 48.053, 66.063, 93.09 111.104, 119.089, 46.031
9	1	—	rs43584258	2.44E-05	16023448	0.36	81.070
9	2	59.57–59.60	rs43602597	3.05E-05	59571491	0.07	119.072
9	1	—	rs41631588	2.14E-05	66997852	0.05	125.132, 157.159, **171.173**
9	1	—	rs41594175	2.39E-05	75484456	0.18	171.173
9	1	—	rs43606008	1.87E-05	86919266	0.38	94.039
9	1	—	rs110950497	2.29E-05	90690991	0.42	109.099, **125.095**
10	2	11.42–11.71	rs42889844	3.35E-06	11707725	0.17	73.027, **75.044**
10	1	—	rs109423971	3.66E-05	16460889	0.36	73.027
10	1	—	rs109393781	7.00E-06	30265387	0.04	83.086
10	1	—	rs43626086	2.89E-05	46923030	0.02	95.017
10	1	—	rs110842319	4.13E-05	86155673	0.27	45.033
11	1	—	rs41569328	2.75E-05	49824565	0.21	83.086
11	1	—	rs41671173	5.30E-07	60150644	0.01	78.001
11	1	—	rs109825961	3.67E-05	78755260	0.02	94.074
11	1	—	rs110622288	3.05E-05	93635833	0.46	94.039
12	2	15.44–15.46	rs29015221	3.23E-05	15462779	0.41	prot_CHEESE_
12	3	56.69–57.90	rs41582811	2.63E-05	57020102	0.49	44.98, 123.117, 1**41.129**, 60.045
12	1	—	rs41620203	2.04E-05	60134810	0.22	105.039
12	2	64.87–65.09	rs110564951	1.31E-05	65092442	0.34	**74.051**, 75.027
13	1	—	rs42283006	4.78E-05	6600008	0.26	49.028
13	1	—	rs41693645	4.70E-05	45112117	0.15	121.122
13	2	72.93–74.05	rs41712280	3.32E-05	74054238	0.25	81.070
14	1	—	rs110323135	2.88E-05	19316702	0.38	**133.123**, 201.184
14	3	26.00–26.95	rs42304786	7.12E-06	26168147	0.04	fat_MILK_, **fat**_**CHEESE**_, 67.058
14	1	—	rs109230206	3.33E-06	44029634	0.26	**121.122**, 147.134
14	1	—	rs29024078	9.75E-06	47996787	0.10	83.086
14	2	81.20–81.31	rs109731285	4.96E-05	81309300	0.35	96.961
15	1	—	rs110215534	2.61E-05	23426546	0.18	33.034
15	1	—	rs43716972	4.71E-05	31193819	0.24	95.017
15	1	—	rs109429918	3.69E-05	55488319	0.01	fat_to_prot_MILK_
15	1	—	rs42530896	3.40E-05	62849070	0.18	113.098
15	1	—	rs41568418	3.09E-05	82388156	0.02	33.034
16	1	—	rs109818696	2.85E-05	12963666	0.06	lactose
16	1	—	rs41798196	9.62E-08	21772991	0.05	**prot**_**CHEESE**_
16	1	—	rs41640566	2.63E-05	57239720	0.04	81.070, 137.132
16	1	—	rs109033026	1.44E-05	60615012	0.03	78.001
16	1	—	rs110311677	4.51E-05	68889225	0.13	**prot**_**CHEESE**_
16	1	—	rs109659498	1.14E-05	71431541	0.25	**84.075**, 119.072
16	1	—	rs41634224	2.54E-05	75232924	0.19	**46.031**, 44.058
16	2	77.29–77.47	rs110300263	1.72E-05	77286529	0.08	75.08, 48.053, 66.063, 83.071, 93.09,93.432, 94.095, 119.072, **93.090**, 94.095
17	1	—	rs109501184	2.86E-05	22820344	0.46	107.066
17	2	33.83–35.38	rs42436295	2.02E-05	33827641	0.04	82.945, **84.942**
17	1	—	rs41577510	2.03E-05	66671839	0.37	57.07
18	3	12.16–13.35	rs41606534	6.58E-06	13346768	0.11	111.119, 121. 122,71.086, 79.075, 121.122, **133.123**, 135.134
18	1	—	rs41867785	2.40E-07	16119985	0.05	71.086, 75.08, 85.101, 48.053, 66.063, 70.064, 79.075, 83.071, 84.075, 93.09, 93.432, 94.095, **95.096**, 111.104, 119.072 119.089, 121.122, 123.076, 133.123, 135.134,136.022 137.101, 139.076, 139.134
18	1	—	rs41574692	3.12E-05	31226355	0.01	92.061
18	1	—	rs110323820	1.75E-06	52739093	0.18	111.119
19	1	—	rs110681423	2.59E-05	48933619	0.02	117.047
19	1	—	rs110985836	4.41E-05	57213764	0.03	113.057
19	6	60.32–63.14	rs41572967	5.78E-06	61630610	0.23	57.033, **57.070**, 117.047
20	2	6.94–6.97	rs43096354	5.41E-06	6935534	0.31	**56.045**, 119.107, 40.027
20	1	—	rs110781147	3.66E-05	13653662	0.44	53.039
20	1	—	rs41631276	3.76E-07	46296840	0.01	Prot_CHEESE_
21	1	—	rs110768892	2.51E-05	11144985	0.01	123.076
21	1	—	rs110582978	8.96E-06	20028323	0.01	57.033
21	3	29.25–30.49	rs41662776	9.19E-06	30485584	0.44	74.051, **125.132**, 123.117, 171.173, 143.143
21	1	—	rs42645046	4.13E-05	36843951	0.11	109.099
21	7	40.72–45.33	rs41984926	8.27E-06	43117013	0.21	131.107,105.071, 147.113
21	1	—	rs110682144		61994597	0.16	44.058
22	1	—	rs41586672	4.43E-05	3126680	0.26	44.022
22	1	—	rs29010379	4.78E-05	20434643	0.14	95.081
22	1	—	rs41608934	3.25E-05	25141851	0.03	84.942
22	1	—	rs41584627	3.67E-05	50926015	0.46	127.073
22	2	56.11–56.92	rs109470329	2.66E-05	56916823	0.16	105.071, **75.08**
23	1	—	rs41633690	3.39E-05	42357362	0.14	Fat_MILK_
23	4	50.14–51.86	rs109579577	9.47E-06	51795496	0.12	82.945, 96.961, 84.942, 100.954, **fat**_**CHEESE**_
24	1	—	rs110415388	3.44E-05	57725812	0.05	66.063
25	1	—	rs110298146	1.13E-05	6968865	0.02	84.942
25	1	—	rs110206231	2.52E-06	16335767	0.43	**39.023**, 40.027
25	1	—	rs109274795	4.88E-05	19082329	0.26	149.045
25	1	—	rs109322753	2.48E-05	24785051	0.01	105.039, **169.044**
25	1	—	rs29012599	2.05E-05	29065778	0.01	95.017
26	1	—	rs110625287	4.58E-05	20597976	0.36	78.001
26	1	—	rs42096562	4.66E-05	26585557	0.01	139.134
26	1	—	rs42704169	2.10E-05	29529492	0.03	113.057
26	1	—	rs110858406	2.35E-05	32030774	0.28	63.044
27	1	—	rs109663833	5.00E-06	42118037	0.03	fat_MILK_
28	1	—	rs109853706	4.70E-05	22792613	0.26	95.049
28	1	—	rs109412394	1.46E-05	28884068	0.19	127.112, **99.081**, 44.022
28	1	—	rs110929815	3.91E-05	39528421	0.30	83.071
28	1	—	rs41600236	4.08E-05	42436188	0.01	105.039
28	1	—	rs110820252	3.89E-05	46146720	0.32	42.010, **75.044**
29	2	26.01–26.24	rs42173924	4.13E-05	26243702	0.31	60.021
29	1	—	rs111007459	4.01E-06	37440150	0.15	57.033
29	1	—	rs42192239	1.31E-06	47269419	0.02	60.045
U^c^	1	—	—	1.04E-05	—	0.27	131.107

#SNP = number of the single nucleotide polymorphisms significantly associated to the trait; Interval: The region on the chromosome spanned among the significant SNP(s) (in Mb); *P*-value (range) = The *P*-value of the highest significant SNP adjusted for genomic control; Top SNP location (bp) = position of the highest significant SNP on the chromosome in base pairs on UMD3.1 (http://www.ensembl.org/index.html); Top SNP MAF = minor allele frequency of the top SNP.

^a^BTA: *Bos taurus* autosome.

^b^MY: milk yield. Cheese volatile compounds are reported as measured mass (m/z). The trait with the highest *P*-value in each genomic region is bolded.

^c^U: Undefined chromosome and position on the genome.

We identified significant associations on BTA4, BTA14, BTA23 and BTA27 for milk fat, with the highest peak corresponding to marker rs42435059 (*P* = 4.43E-06) located at 39,244,447 on BTA4. We also identified significant associations for milk protein and milk yield on BTA6, the highest signal being associated with milk protein and corresponding to the marker rs110239739 (*P* = 1.32E-07) located at 84,689,991 bp. Only 1 SNP (rs109429918) was significant for the fat-to-protein ratio and this was located on BTA15 at 55,488,319 Mbp. A significant association was found for lactose on BTA16 and corresponded to rs109818696 located at 12,963,666. Significant SNPs for cheese fat were mapped on BTA 14 (~26.17 Mbp) and BTA23 (~42.36 Mbp). We detected very high peaks for cheese protein on BTA16 and BTA20, corresponding to markers rs41798196 located at 21,772,991 on BTA16 (*P* = 9.62E-08) and rs41631276 located at 46,296,840 on BTA20 (*P* = 3.76E-07). We found other significant associations for cheese protein on BTA12 at ~15.45 Mbp.

Regarding cheese VOCs, we detected the strongest signals on BTA11 and BTA18. Marker rs41671173 located at 60,150,644 bp on BTA11 was significant for the spectrometric peak at *m/z* 78.001 (*P* = 5.30E-07). We detected another strong signal at 16,119,985 bp on BTA18 and corresponded to marker rs41867785, which was associated with the peak at *m/z* 135.134 (*P* = 1.10E-07). Overall, this marker was significant for 24 spectrometric peaks, three of which were tentatively associated with butan-1-ol/pentan-1-ol, heptan-1-ol^9,11^ *m/z* 75.080 (*P* = 1.97E-06), 3-methyl-1-butanol/3-methyl-3-buten-1-ol/pentan-1-ol *m/z* 71.086 (*P* = 1.20E-05) and hexan-1-ol/hexan-2-ol *m/z* 85.101 (*P* = 1.61E-05). The largest regions of consecutive SNPs were located on BTA6 (~81.65–88.07 Mbp) and BTA21 (~40.72–45.33 Mbp). The spectrometric peaks with the highest number of significant SNPs were those associated with ethyl pentanoate (ethyl valerate)/ethyl-2-methylbutanoate/ethyl-3-methylbutanoate (ethyl isovalerate)/heptanoic acid *m/z* 131.107 (6), *m/z* 149.045 (5), *m/z* 48.053 (5), *m/z* 66.063 (5), the peaks associated with butan-1-ol/pentan-1-ol, heptan-1-ol *m/z* 75.080 (5), *m/z* 84.942 (5), *m/z* 105.039 (5), *m/z* 117.047 (5), *m/z* 119.072 (5), *m/z* 121.122 (5) and *m/z* 169.044 (5). The chromosomes with the highest number of significant associations were BTA1 (10), BTA3 (9), BTA4 (11), BTA6 (16), BTA16 (9) and BTA21 (14).

**Figure 1:**
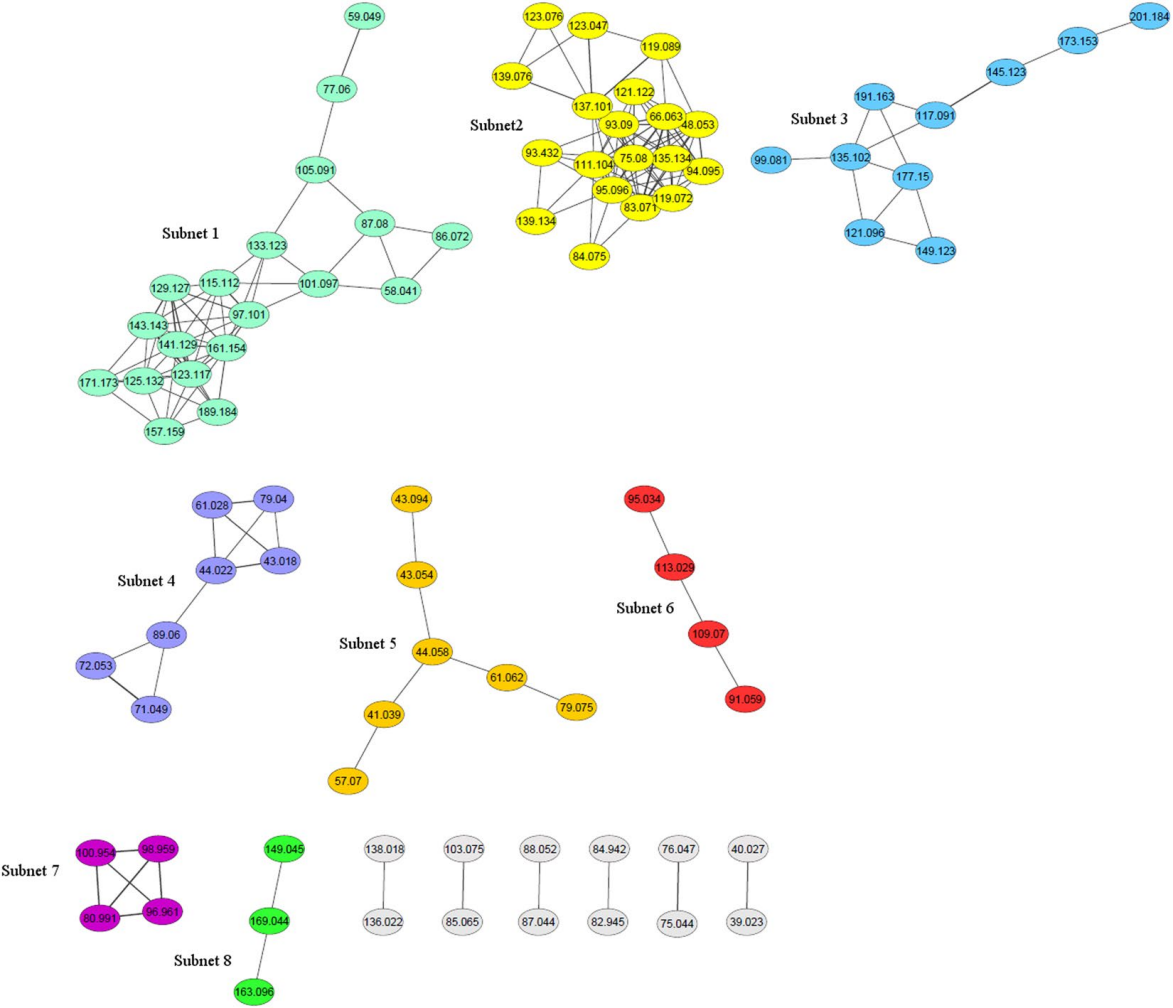
Similarity network among cheese volatile compounds generated using ExpressionCorrelation. The nodes corresponded to cheese VOCs and the edges represented the similarity between vectors of the additive effects of all SNPs. Only correlations with *r* > |0.80| and *P* < 0.01 are represented. Eight sub-networks of ≥3 nodes were identified which contained significantly dense clusters of VOCs (*P* < 0.05) detected by ClusterOne. The width of the edge indicates the value of the correlation; a wider edge corresponds to a higher correlation in absolute value.

Based on the similarity matrix generated with ExpressionCorrelation, we identified 8 sub-networks represented by ≥3 nodes (Fig. 1), within which ClusterOne identified 12 densely connected clusters (*P* < 0.05; Supplementary Table S2). Two clusters were detected in sub-network 1: one with 13 nodes, which included some spectrometric peaks tentatively associated with aldehydes and/or ketones, i.e. hexan-1-one/hexan-2-one/hexanal *m/z* 101.097, heptan-2-one *m/z* 115.112, octan-1-one *m/z* 129.127 and nonan-2-one *m/z* 143.143; the other with 7 nodes, which included some spectrometric peaks associated with aldehydes, ketones or alcohols, i.e. propan-2-one (acetone) *m/z* 59.049, 1,2-pentanediol *m/z* 105.091 and 2-methylbutanal/3-methylbutanal/pentan-2-one *m/z* 87.080. A cluster of 16 nodes was significant in subnetwork 2, which included the spectrometric peak associated with butan-1-ol *m/z* 75.080. Sub-network 3 comprised 2 clusters with 7 nodes, which contained some spectrometric peaks associated with esters and/or alcohols, i.e. ethyl hexanoate/octanoic acid *m/z* 145.123, ethyl butanoate/ethyl-2-methylpropanoate (ethyl isobutyrate) *m/z* 117.091 and hexanoic acid *m/z* 99.081. Two clusters were detected in sub-network 4: the first with 4 nodes, including the spectrometric peak associated with acetates/acetic acid *m/z* 61.028; the second also included the spectrometric peaks tentatively identified as 3-hydroxy-2-butanone (acetoin) *m/z* 89.060 and butanoic acid *m/z* 71.049. Two clusters with 5 nodes were significant in sub-network 5, which included spectrometric peaks associated with the alkyl fragment *m/z* 43.054, *m/z* 41.039 and *m/z* 57.070. Sub-network 6 contained a significant cluster which included the spectrometric peak associated with 2,6-dimethyl pyrazine *m/z* 109.070. Sub-networks 7 and 8 contained clusters of 4 and 3 nodes, respectively, neither of which included any tentatively identified spectrometric peaks.

### Pathway analyses

Of the total 37,568 SNPs used in this study, 17,006 were located 15 kb up- or down-stream of the coding regions. An average of around 900 genes were significant (*P* < 0.05) for the peaks tentatively associated with cheese VOCs. We carried out pathway analyses to shed light on the biological role of these genes and to identify potentially overrepresented pathways or molecular functions that might help explain the variability in the cheese volatilome.

**Figure 2:**
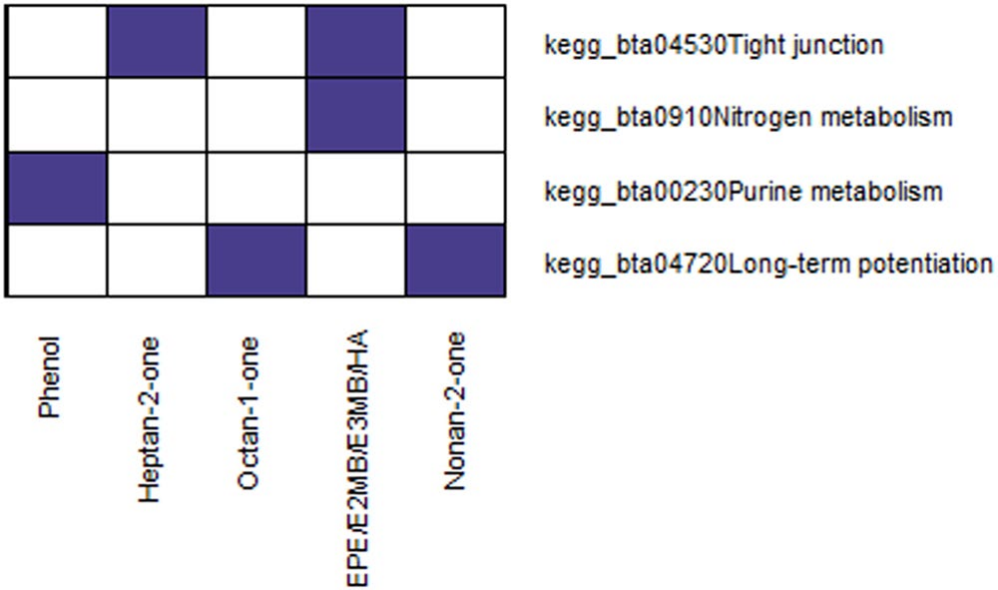
Significantly enriched KEGG pathways using genes associated to spectrometric peaks with a tentative identification from PTR-ToF-MS analysis of model cheeses. Only the traits showing significantly enriched terms are reported (FDR < 0.05). EPE_E2MB_E3MB_HA: Ethyl pentanoate (ethyl valerate)-Ethyl-2-methylbutanoate-Ethyl-3-methylbutanoate (ethyl isovalerate)-Heptanoic acid.

Overall, pathways of 5 of the 45 tentatively identified compounds were significantly enriched (FDR < 0.05) (Fig. 2, Supplementary Table S3). Results showed that purine metabolism was enriched for the peak associated with phenol *m/z* 95.049 (FDR = 0.00017), while the tight junction pathway was overrepresented for the spectrometric peaks associated with heptan-2-one^20^ *m/z* 115.112 (FDR = 0.00013) and ethyl pentanoate (ethyl valerate)/ethyl-2-methylbutanoate/ethyl-3-methylbutanoate (ethyl isovalerate)/heptanoic acid *m/z* 131.107. Furthermore, the nitrogen metabolism pathway was significantly enriched for the peak associated with ethyl pentanoate (ethyl valerate)/ethyl-2-methylbutanoate/ethyl-3-methylbutanoate(ethyl isovalerate)/heptanoic acid (FDR = 0.00019) *m/z* 131.107. Finally, the long-term potentiation pathway was enriched for the peaks associated with octan -1–one *m/z* 129.127 and nonan-2-one *m/z* 143.143 (FDR = 0.00023 and FDR = 0.00024, respectively).

## Discussion

### GWAS analysis

In recent years, there has been growing concern about food quality and safety from both the demand and the supply sides. Given that flavour attributes play a crucial role in cheese quality^21^, better knowledge of the key flavour components and pathways involved in the development and characterisation of cheese VOCs would provide a useful basis for defining cheese-making procedures more precisely, and improving cheese sensory characteristics. There is also increasing interest in the authentication of traditional cheeses with EU protected designation of origin classification, which are often linked to local breeds and help maintain farm animal biodiversity^22^. In this study, therefore, we first sought to investigate whether the cow’s genome organisation significantly impacts on the cheese volatilome, and possibly cheese flavour.

Although plenty of GWAS studies for milk production traits^13,23,24^ and cheese-making properties^25,26^ in dairy cows have now been published, to our knowledge none has focused on identifying the genomic regions associated with cheese composition and quality traits. Despite the lack of GWAS analyses for cheese VOCs, the estimates of genomic heritability found in this study confirm previous findings supporting the existence of an exploitable genetic variation in cheese VOCs^11^. The main pathways involved in the formation of cheese VOCs are glycolysis (metabolism of lactose, lactate and citrate), lipolysis (and metabolism of fatty acids) and proteolysis (and catabolism of amino acids)^3^. Accordingly, our GWAS analyses revealed a contribution of cow’s genes related to protein, fat and carbohydrate metabolism.

### Protein metabolism

A region of 9 SNPs on BTA6 covered the cluster of casein genes (~87.14–87.38 Mbp) and showed significant association with 12 traits, including milk protein. In particular, 4 spectrometric peaks - *m/z* 85.029, *m/z* 149.045, *m/z* 163.096 and *m/z* 169.044 - were associated with the marker rs41567942, which was located 0.4 Mb from the gene encoding for k-casein *(CSN3*), which is essential for milk coagulation and therefore largely influences milk coagulation properties^27^. Moreover, the marker rs29001782 was located on BTA6 at 4 kb from *GNRHR*, which signalling pathway has been shown to play a role in controlling milk protein synthesis and metabolism^17^. Interestingly, the markers rs110300263 and rs111018457, which had significant associations with 10 traits, were located in the region at ~77.29–77.47 Mbp on BTA16, which was close to a quantitative trait locus (QTL) for the milk protein and k-casein percentages^28^. Two markers, which were associated to *m/z* 56.045 and *m/z* 119.107 (rs43096354) and *m/z* 40.027 (rs42353243), mapped on BTA20 at ~0.3 Mb from *FAM169A* which has been suggested to be a key regulator of milk protein synthesis in dairy cattle^17^. Additionally, the region of 7 SNPs on BTA21 included a known QTL for milk fat and protein yield and percentage from the Cattle QTL database information^28^.

### Fat metabolism

The contribution of fatty acid metabolism to cheese VOCs is corroborated by several significant associations. For instance, rs43283349, which was significant for 3-methylbutyl butanoate (isoamyl butyrate)/nonanoic acid *m/z* 159.138, was located on BTA1 at ~0.1 Mb from *AGPAT3*, a positional candidate gene for milk FA^29^. The marker rs110986676, which was located on BTA6 and was significant for *m/z* 116.078, corresponded to an intron variant of *SCD5* which was associated to variation in milk FA composition in dairy cattle^16,30^. The marker rs110681423, which was associated with *m/z* 117.047, was located on BTA19 at ~0.2 Mb from *GH1* which has been put forward as candidate gene for milk fat percentage and fat composition^12,31^. The marker rs110858406, associated to *m/z* 63.044, mapped on BTA26 at ~0.9 Mb from *GPAM* which is involved in the regulation of milk fat synthesis and composition in dairy cattle^32,33^. Finally, rs110820252, which had significant associations with the spectrometric peaks associated with the alkyl fragment *m/z* 42.01 and propanoic acid/ propanoic ester *m/z* 75.044 mapped on BTA28 within 2 kb 5′ to *AGT*, which is the sole precursor of all angiotensin peptides. Interestingly, the renin-angiotensin system is believed to impact body-fat storage as well as lipid and carbohydrate metabolism^34,35^.

### Carbohydrate metabolism

A significant association was found between rs110002748 and *m/z* 117.047, which mapped on BTA8 at ~1 Mb from *B4GALT1*. This gene encodes an enzyme that participates in glyconjugation and lactose biosynthesis, which occurs exclusively in the mammary gland^36^. An increase in the expression of *B4GALT1* was observed in transition milk samples, and is reflected in an increase in lactose biosynthesis during the earlier stages of lactation^37^. The high signal detected on BTA11 (rs41671173) was located on BTA11 at ~0.5 Mb from *B3GNT2*, which synthesizes a unique structure known as poly-N-acetyllactosamine (polyLacNAc), a linear carbohydrate polymer composed of alternating N-acetylglucosamine and galactose residues^38^. This SNP explained ~60% of additive genetic variance for *m/z* 78.001. Finally, the high signals on BTA18 corresponded to the marker rs41867785, which is annotated as an intron variant of *PHKB*. This gene has been associated with the carbohydrate metabolic process, the generation of precursor metabolites and energy, and energy reserve^39^.

### Correlations among VOCs based on SNP additive effects

A greater level of detail concerning the shared genomic basis of cheese VOCs might form the basis for more accurate prediction models to be developed in the context of genomic selection for possible modulation of cheese flavour. In a previous work, we estimated the genetic relationships among cheese VOCs based on pedigree information^11^. Here, we used ExpressionCorrelation to calculate pairwise correlations between VOCs based on the SNP additive genetic effects, and we clearly identified groups of VOCs sharing a common behaviour. Having tentatively identified some compounds, we sought to associate the largest sub-networks to biochemical pathways and possibly associated flavour notes. Sub-network 1 contained mostly ketones and aldehydes and might, therefore, represent catabolism of amino acids and fatty acids. Branched-chain aldehydes originate from AA degradation, in particular 2-methylbutanal from isoleucine and 3-methylbutanal from leucine^40^, while ketones can be produced from β-ketoacids derived from β-oxidation of fatty acids^41^. Green/fruity/floral notes are mostly associated with the compounds included in this group^20,40,42^. The reaction between free fatty acids and alcohols from lactose and AA degradation yield esters^43^, common cheese VOCs, and this pathway might be represented in sub-network 3, including the spectrometric peaks associated with hexanoic acid, ethyl hexanoate/octanoic acid and ethyl butanoate/ethyl-2-methylpropanoate (ethyl isobutyrate). Most esters (e.g. ethyl butanoate, ethyl hexanoate, ethyl-2-methylpropanoate) are associated with the sweet, fruity and floral characteristics of cheese^44–46^. Finally, sub-network 4 might represent the glycolysis pathway, and, in particular, lactate or citrate metabolism, since it included the spectrometric peaks associated with the acetate ester fragment/acetic acid, 3-hydroxy-2-butanone(acetoin) and butanoic acid. Lactose is metabolised by starter bacteria, mostly through the glycolytic pathway, into lactate, which might be further metabolised into acetate by lactococci or into butyrate by *Clostridium* sp.^47^. Acetate is also the main flavour compound originating from citrate metabolism as well as acetoin^47,48^. Cheesy, rancid and sour milk notes are associated with 3-hydroxy-2-butanone(acetoin) and butanoic acid^45,49^, while acetic acid has a typical vinegar odour^50^.

### Pathway analysis

Standard GWAS analysis allows individual loci and genes likely to play a role in controlling the investigated traits. However, it lacks the power to establish whether the detected genes act in cooperation as part of a complex network to control specific biological functions. We therefore carried out pathway analyses to prioritize genes in associated loci that are part of the biological pathways and processes potentially contributing to the cheese volatilome.

These pathway analyses confirmed the importance of proteolysis and amino acid metabolism for the formation of cheese VOCs (i.e. nitrogen and purine metabolism). Phenol in cheese originates from the metabolism of protein (casein) and, in particular, from the catabolism of tyrosine^3^. Besides sugar and fat metabolism, amino acid metabolism also provides substrates for ester formation, which might explain the enrichment of nitrogen metabolism for the spectrometric peaks associated with ethyl pentanoate (ethyl valerate)/ethyl-2-methylbutanoate/ethyl-3-methylbutanoate(ethyl isovalerate)/heptanoic acid *m/z* 132.109. The tight junction pathway was enriched for the spectrometric peaks associated with heptan-2-one and ethyl pentanoate (ethyl valerate)-ethyl-2-methylbutanoate-ethyl-3-methylbutanoate (ethyl isovalerate)-heptanoic acid. In the mammary gland, the tight junction (TJ) state is closely linked to milk secretion^51^, as they are involved in the transcellular transport of lactose and K+ to the extracellular fluid, while Na+ and Cl− are transported to the milk^52^. TJ integrity is compromised during mammary involution and also as a result of mastitis and periods of mammary inflammation^53^. Among the genes identified within this pathway, we found three protein kinase C (PKC) family members: alpha (*PRKCA*), beta (*PRKCB*) and epsilon (*PRKCE*). Several PKC inhibitors affect both the assembly and disassembly of TJs, which means that PKCs may regulate the dynamics of TJ formation^54^. Interestingly, this pathway was enriched for the energy of the curd as a percentage of the energy of the milk processed, which is an indicator of cheese-making efficiency^55^. Finally, enrichment of the long-term potentiation pathway for the spectrometric peaks associated with two ketones, octan-1-one and nonan-2-one, might be connected to their biosynthetic pathway, which is related to fatty acid metabolism; indeed, this pathway was significantly overrepresented in a recent GWAS and pathway-based analysis of milk fatty acids in dairy cows^16^. Moreover, this pathway contained several genes coding for glutamate ionotropic receptors (GRI), including *GRIA1*; it is of note that previous findings assigned to this gene a significant SNP for C14:1^15^.

In our study, we exploited the potential of PTR-ToF-MS to provide detailed spectral information to characterise food quality and authentication, and this was integrated with the genomic and biological information provided by GWAS and pathway analyses. Results obtained increase our understanding of the metabolic pathways and biological functions likely involved in the formation of cheese VOCs, providing unprecedented insights into the potential contribution of the cow’s genes to cheese flavour. A more effective approach might be to more accurately identify compounds using PTR-MS and to improve the quality of cattle genome annotations.

## Methods

### Ethics statement

The cows in the current study belonged to commercial private herds and were not subjected to any invasive procedures. Milk and blood samples were previously collected during routine milk recording coordinated by technicians from the Breeders’ Association of Trento Province (Italy), hence certified by the local authority.

### Phenotypes and genotypes

Individual milk samples were collected from 1,075 Italian Brown Swiss cows from 72 commercial herds located in the Alpine province of Trento (Italy). Details of the animals used in this study and the characteristics of the area are reported in Cipolat-Gotet *et al*.^56^ and Cecchinato *et al*.^57^ Gross milk composition was measured using a MilkoScan FT6000 (Foss Electric A/S Hillerød, Denmark). Model cheeses were manufactured from the raw milk of individual cows, as described in detail in Cipolat-Gotet *et al*.^56^. We used a commercial starter culture at a concentration 8 times higher than recommended in order to reduce the acidification time to 90 min and minimise the role of milk microflora. After ripening (60d), the model cheeses were weighed and analysed for fat and protein contents using a FoodScan apparatus (Foss Electric, Hillerød, Denmark). The headspace gas of each model cheese (n = 1,075) was measured with a commercial PTR-ToF-MS 8000 instrument supplied by Ionicon Analytik GmbH, Innsbruck (Austria), as described in detail in Bergamaschi *et al*.^10^ Internal calibration and peak extraction was performed according to the procedure described by Cappellin *et al*.^58^ Absolute headspace VOC concentrations, expressed as parts per billion by volume (ppb_v_), were estimated using the formula described by Lindinger *et al*.^59^ Given that the distribution of all spectrometric peaks showed a strong positive skewness, the data were transformed: the fraction of each peak plus one was multiplied by 10^6^ and expressed as a natural logarithm to obtain a Gaussian-like data distribution. After filtering out all peaks below a threshold of 1 ppb_v_ and interfering ions, 240 spectrometric peaks remained for the analyses. The fragmentation pattern of 61 relevant compounds, representing 78.0% of the total spectral intensity of the compressed data set without interfering ions, were retrieved from available GC-MS data on the same model cheeses^10^ and from the literature^60–62^. Isotope removal (r > 0.95, *P* < 0.001) yielded 173 spectrometric peaks, of which 45 were tentatively associated with VOCs.

The Illumina BovineSNP50 v.2 BeadChip (Illumina Inc., San Diego, CA) was used to genotype 1,152 cows (blood samples were not available for all the phenotyped animals). Quality control excluded markers with call rates >95%, with minor allele frequencies >0.5%, and without extreme deviation from Hardy-Weinberg equilibrium (*P* > 0.001, Bonferroni corrected). After filtering, 1,011 cows and 37,568 SNPs were retained for subsequent analyses.

### Genome-wide association study

Genome-wide association analyses (GWAS) were conducted using single-marker regression and the three-step Genome-wide Association using the Mixed Model and Regression-Genomic Control (GRAMMAR-GC) approach^63^ implemented in the GenABEL R package^64^. In the first step, an additive polygenic model with a genomic relationship matrix is fitted; secondly, the residuals obtained from this model are regressed on the SNPs to test for associations; in the third step, genomic control corrects for the conservativeness of the procedure^65^. The polygenic model was:

1 $${\bf{y}}={\bf{X}}\beta +{\bf{a}}+{\bf{e}},\,$$

where **y** is a vector of the observed response (milk fat, protein and fat-to-protein ratio; cheese fat and protein; cheese VOCs); *β* is a vector with the fixed effects of (i) days in milk of the cow (classes of 30 days each), (ii) the parity of each cow (classes of 1, 2, 3, ≥4), and (iii) the herd-date effect (n = 72); **X** is an incidence matrix connecting each observation to specific levels of the factors in *β*. The two random terms in the model were the animal and the residuals, which were assumed to be normally distributed as ${\boldsymbol{a}} \sim N(0,{\bf{G}}{\sigma }_{g}^{2})$ and ${\boldsymbol{e}} \sim N(0,{\bf{I}}{\sigma }_{e}^{2})$, where **G** is the genomic relationship matrix, **I** is the identity matrix, ${\sigma }_{g}^{2}$ is the additive genomic variance and ${\sigma }_{e}^{2}$ the residual variance. The **G** matrix was built in GenABEL^64^ using identity-by-state coefficients. We adopted a threshold of *P* < 5 × 10^−5^ to declare significant SNPs^66^.

The proportion of genomic variance explained by the SNPs was calculated as 2*pqa*^2^, where *p* and *q* were the allele frequencies and *a* was the allele substitution effect. Model (1) was also used to estimate the variance components and the genomic heritability of the traits based on the genomic relationship matrix. Heritability was estimated as ${h}^{2}=\frac{{\sigma }_{g}^{2}}{{\sigma }_{g}^{2}+{\sigma }_{e}^{2}}$.

The results of the GWAS analysis without filtering for the *P*-value threshold were used to build a matrix of row-wise SNPs (n = 37,568) and column-wise phenotypes (i.e. cheese VOCs, n = 173) in which the value in the cell corresponded to the SNP additive effect. This matrix was fed into the ExpressionCorrelation plugin of Cytoscape^67^ to create a correlation matrix of pair-wise Pearson correlations between phenotypes based on the effect across all the SNPs included in the analysis. Only the high-confidence correlations with *P* < 0.01 and >|0.80| were selected. A similarity network was generated by ExpressionCorrelation, where the nodes corresponded to the phenotypes and the edges represented the similarity between vectors of the additive effects of all SNPs. This network was analysed with the ClusterOne plugin of Cytoscape^68^ to identify significantly dense clusters of VOCs (Mann-Whitney test, *P* < 0.05).

### Gene-set enrichment and pathway analyses

Pathway analyses were carried out on the tentatively identified spectrometric peaks (n = 45) to shed light on the biological functions underlying the synthesis and/or metabolism of cheese VOCs. As detailed in Dadousis *et al*.^55^, the GWAS results were filtered for significance with a *P*-value < 0.05 to identify “relevant” and “non-relevant” SNPs. Using the BiomaRt R package^69,70^, we assigned “relevant” SNPs to genes if they were located within the gene or within 15 kb up- or down-stream of the gene^71^ based on the Ensembl *Bos taurus* UMD 3.1 assembly. This made it possible to also capture those SNPs that are missed by standard GWAS, due to its stringent significance threshold, but that may help explain the variability in the observed phenotypes, which may play a role in organised pathways or biological functions. The Kyoto Encyclopaedia of Genes and Genomes (KEGG)^72^ and the Gene Ontology (GO) databases^73^ were used to define the functional categories associated with the gene sets. To avoid testing broad or narrow functional categories, only GO and KEGG terms with >10 and <1000 genes were considered. A Fisher’s exact test was used to test for overrepresentation of functional categories (FDR < 0.05). The gene-set enrichment analysis was performed with the R package goseq^74^.

## Electronic supplementary material

Supplementary Dataset 1

Supplementary Dataset 2

Supplementary Dataset 3
